# Synthesis of Some Green Dopants for OLEDs Based on Arylamine 2,3-disubstituted Bithiophene Derivatives

**DOI:** 10.3390/molecules181114033

**Published:** 2013-11-13

**Authors:** Mi-Seon Song, Quynh Pham Bao Nguyen, Chang-Hyun Song, Duckhee Lee, Kyu Yun Chai

**Affiliations:** The Division of Bio-Nanochemistry, The College of Natural Sciences, the Wonkwang University, Iksan City, Chonbuk, 570-749, Korea

**Keywords:** green dopants, bithiophene, 2,2-diphenylvinyl, 9-phenylcarbazole, triphenylamine, *N,N'*-di-(*p*-tolyl)benzeneamine

## Abstract

A series of green dopants based on 2,2-diphenylvinyl end-capped bithiophene and three different arylamine moieties (9-phenylcarbazole, triphenylamine, and *N,N’*-di-(*p*-tolyl)benzeneamine) were successfully synthesized by the Suzuki and Wittig coupling reactions. The photophysical properties of these compounds are reported. The strongest PL emitting compound with the 9-phenylcarbazole moiety has been used for fabricating an OLED device with good overall performance.

## 1. Introduction

Organic light-emitting diodes (OLEDs) have attracted increasing interest over the last few years because of their potential to achieve low-cost, full-color and flat-panel displays [1]. One of the key developments in OLED display technology can be attributed to the discovery of the guest-host doped emitter system in which a single host material with optimized transport and luminescent properties may be used together with a variety of highly fluorescent guest dopants, leading to its electro-luminescence of desirable hues with very high efficiencies [2]. In addition, the doped emitter system enhances its operational stability by transferring the electrogenerated exciton to the highly emissive and stable dopant site, thus minimizing its possibility for nonradiative decay [3]. Consequently, many red, green, and blue fluorescent dopants have been extensively studied to obtain full color displays [2]. For example, green dopants based on coumarin, quinacridone, quinolone, quinnoxaline, and carbazole derivatives with promising electroluminescent efficiencies have been exploited [3,4].

Oligothiophenes with well-defined structures have been widely exploited in the field-effect transistors and light emitting diodes because they showed high conductivity and unique optical properties which could be finely tuned by their chain length and terminal substituents [5,6,7,8,9]. The strategy of introducing different groups to cap oligothiophenes has been used to diversify the structure of a conjugated backbone, offering new possibilities of efficient electronic and optical properties. As previously reported, oligothiophenes terminated with phenyl or biphenyl groups exhibit interesting electroluminescent behavior and oligothiphones with terminal groups bearing diaryamino fuctional groups have been used as efficient emitters and potential hole-transporting materials. Other oligothiophene end-capping groups such as diaryboryl, pyridyl, diphenylphosphine, and charge-transfer capable groups have also been documented [5,10,11,12,13]. 

**Figure 1:**
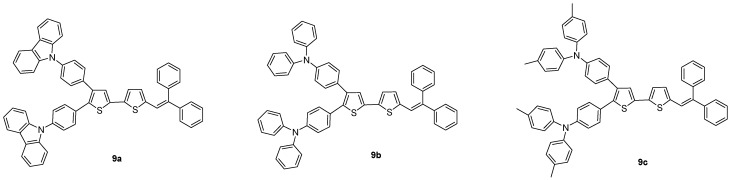
Structures of designed green dopants **9**.

Presented herein are the design, synthesis and characterization of a new class of the green dopants based on arylamine 2,3-disubstituted bithiophene derivatives end-capped with the 2,2-diphenylvinyl group (Figure 1). The arylamine substituents and diphenylvinyl group on the bithiophene core were expected to enhance light emitting efficiency through the extended conjugated structures and to improve the charge transport properties. In addition, the two phenyl rings as end-capping groups would force the structures to twist due to steric hindrance and thus prevent self-quenching by molecular aggregation [8,9,14,15,16]. 

## 2. Results and Discussion

**Scheme 1:**
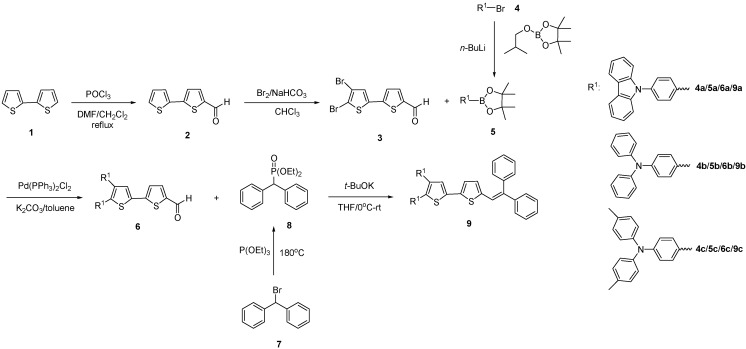
Synthesis of green dopants **9**.

The designed green dopants **9** based on arylamine 2,3-disubstituted bithiophene derivatives end-capped with the 2,2-diphenylvinyl group were prepared in four steps from the commercially available bithiophene (**1**, Scheme 1). First, bithiophene (**1**) was formylated with the Vilsmeier reagent (POCl_3_/DMF) in dichloromethane following a reported procedure to form compound **2** [17], which was then brominated with Br_2_/NaHCO_3_ to produce compound **3** in 80% yield. Pd-catalyzed Suzuki coupling reactions using Pd(PPh_3_)_2_Cl_2_/NaHCO_3_ in toluene between compounds **3** and **5** proceeded very well to give 80%–85% yields of compounds **6**. Finally, the designed green dopants **9** end-capped with the 2,2-diphenylvinyl group were obtained in moderate yields (53%–58%) by the Wittig coupling reactions of compounds **6** and **8** in the present of *t*-BuOK. The three green dopants **9a**–**c** were identified and characterized by NMR, IR, UV-vis, PL spectroscopies and energy levels HOMO-LUMO before the fabrication of OLED devices. 

**Figure 2:**
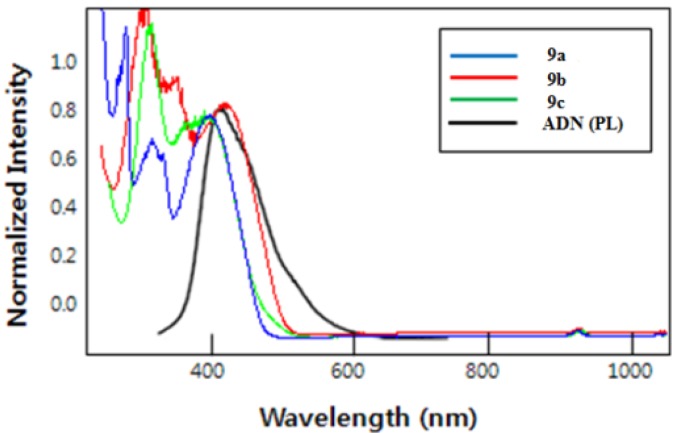
UV spectra of green dopants **9**.

**Figure 3:**
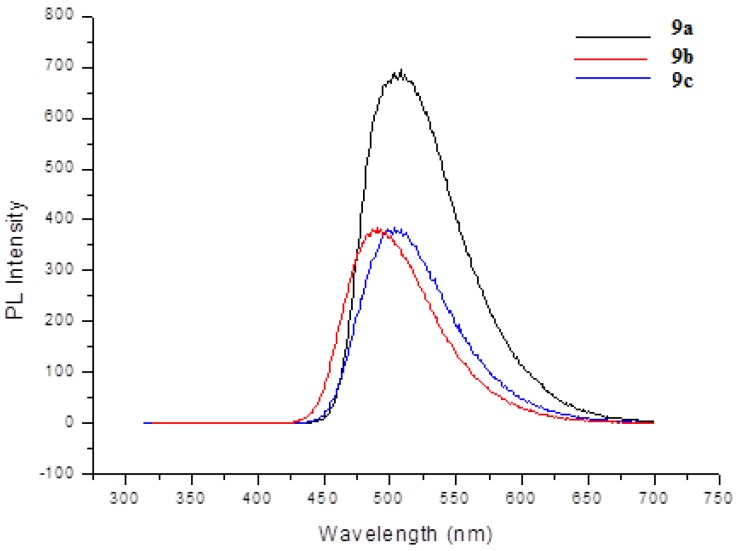
PL spectra of green dopants **9**.

Figure 2 and Figure 3 present the UV-vis and PL spectra of the green dopants **9** in dichloromethane with the results summarized in Table 1. The UV-vis absorption peaks of **9a**–**c** were observed at 402, 409, and 376 nm, respectively. As seen in Figure 2, the absorption spectra of **9a**–**c** overlapped with the emission spectra of the common host material 9,10-di(2-napthyl)anthracene (ADN). Interestingly, the absorption spectra of **9b** overlapped more effectively with the emission spectra of ADN than that of **9a** and **9c**. 

These observations imply that **9a**–**c** could accept energy from the ADN host material by a Förster-type energy transfer and ADN acted as a good host in the OLED devices using **9a**–**c** as dopants. Particularly, a Förster-type singlet energy transfer from ADN host to **9b** would be more effective than that of **9a** and **9c**. The emission peaks of **9a**–**c** were observed in the green region at 507, 491, and 502 nm, respectively (Figure 3 and Table 1). Notably, with the same bithiophene core, compound **9b** with triphenylamine moiety turned out to have a slight blue shift, leading to emit the bluish green color and compound **9a** with 9-phenylcarbazole moiety exhibited extremely higher PL intensity than compounds **9b** and **9c**. The fluorescence quantum yields of **9a**–**c** in chloroform were 0.94, 0.44, and 0.45, respectively [18].

**Table 1:** Photophysical properties of green dopants **9**.

Compound	UV λ_max_	PL λ_max_	E _ox_	HOMO (eV) ^d^	LUMO (eV) ^d^	E_g_	Ф_f_^e^
	(nm) ^a^	(nm) ^a^	(eV) ^c^			(eV)	
NPB	- ^b^	- ^b^	0.55	5.40	2.50	2.90	- ^b^
9a	402	507	0.76	5.61	2.97	2.64	0.94
9b	409	491	0.58	5.43	2.88	2.55	0.44
9c	376	502	0.49	5.34	2.58	2.76	0.45

^a^ In dichloromethane (ca. 1 × 10^−6^ M); ^b^ Not determined; ^c^ Oxidation potential relative to Ag/AgCl electrode; ^d^ HOMO = (E_ox_ + 4.85) eV; LUMO = (HOMO − E_g_) eV; ^e^ In chloroform with coumarin 153 as a standard.

In addition, from the cyclovoltammetric measurements and the bandgap energies (E_g_) which were estimated from the onset of absorption, the energy of the highest occupied molecular orbital (HOMO) and that of the lowest unoccupied molecular orbital (LUMO) of the green dopants **9a**–**c** were determined and compared with those of *N,N'*-di(naphthalen-1-yl)-*N,N’*-diphenyl-benzidine (NPB), one of the most widely used hole-transport material in OLEDs (Table 1). The HOMO/LUMO energy levels of compounds **9a**–**c** were 5.61/2.97, 5.43/2.88, and 5.34/2.58, respectively, and their calculated HOMO-LUMO energy gaps (E_g_) were 2.64, 2.55, and 2.76, respectively. For efficient device operation it is necessary to have HOMO and LUMO levels of the emitter suitably aligned with the HOMO and LUMO of hole-transport and electron-transport materials. As seen in Table 1, compounds **9a**–**c** satisfied the energy level conditions to be potentially used as green dopants in OLED devices.

**Figure 4:**
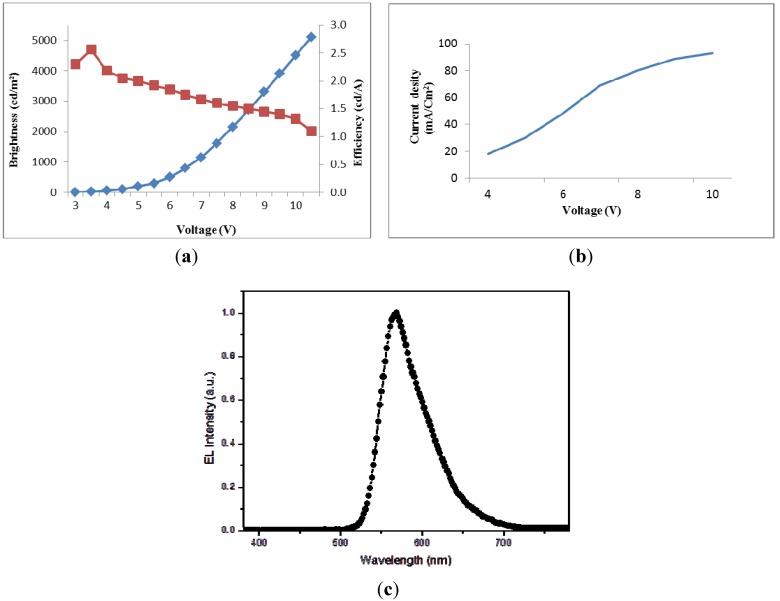
Device characteristics of the OLED with the configuration of ITO/NPB (10 nm)/**9a** (30 nm)/Bebq_2_ (10 nm)/LiF/Al.

Finally, compound **9a** with the strongest PL emission was selected as a potential dopant for fabricating an OLED device with the configuration of ITO/NPB (10 nm)/**9a** (30 nm)/Bebq_2_ (10 nm)/LiF/Al. A yellowish green color (CIE = 0.42, 0.54) with a maximum emission peak at 568 nm was emitted. The brightness, luminous efficiency and current density were shown in Figure 4. The device exhibited good overall performance with a maximum brightness and luminous efficiency of 5,100 cd/m^2^ and 2.56 cd/A, respectively.

## 3. Experimental

### 3.1. General Procedures

All reagents and solvents were obtained from commercial suppliers (Aldrich, Seoul, Korea and TCI Chem. Co., Seoul, Korea) and were used without further purification. ^1^H- and ^13^C-NMR spectra were recorded on a JEOL JNM-ECP FT-NMR spectrometer operating at 500 and 125 MHz, respectively. IR spectra were measured on a Shimadzu Prestige-21 FT-IR spectrophotometer. The samples were prepared as a KBr pellet and scanned against a blank KBr pellet background at a wave number ranging from 4000 to 400 cm^−1^. UV-vis absorption spectra were recorded on a Scinco S-3100 spectrophotometer while photoluminescence (PL) spectra were measured on a CARY Eclipse Varian fluorescence spectrophotometer. The HOMO levels were calculated from the oxidation potentials, while the LUMO levels were calculated based on the HOMO levels and the lowest-energy absorption edges of the UV-vis absorption spectra.

### 3.2. Synthesis

Compounds **2**, **5** and **8** were known and synthesized by following the methods previously reported in the corresponding literature [17,19,20].

#### 3.2.1. Synthesis of 5-(4,5-dibromothiophen-2-yl)thiophene-2-carbaldehyde (**3**)

To a stirred solution of 5-(thiophen-2-yl)thiophene-2-carbaldehyde (**2**, 1 g, 5.15 mmol) in chloroform (30 mL) under an Ar atmosphere was added Br_2_ (2.4 g, 5.15 mmol) and NaHCO_3_ (0.43 g, 5.15 mmol). The reaction mixture was refluxed for 4h, cooled to room temperature, filtered, and then evaporated to remove the solvent. The obtained crude product was purified by column chromatography to give the requisite product **3**. Yield: 80%; yellow solid; m.p: 124–126 °C; FT-IR: ʋ_max_ 1672, 1449, 1422, 1264, 730 cm^−1^; ^1^H-NMR (CDCl_3_) δ 9.82 (s, 1H), 7.65 (s, 1H), 7.32 (d, *J* = 5.0 Hz, 1H), 7.09 (d, *J* = 5.0 Hz, 1H); ^13^C-NMR (CDCl_3_) δ 181.6, 140.9, 140.1, 134.9, 130.5, 128.8, 116.3, 108.5.

#### 3.2.2. Typical Procedure for Synthesis of Compounds **6**

To stirred solutions of compound **3** (1 g, 2.8 mmol) and compounds **5** (7 mmol) in toluene (30 mL) were added Pd(PPh_3_)_2_Cl_2_ (0.1 g, 0.14 mmol) and K_2_CO_3_ (3 g, 21 mmol) in water (10 mL). The reaction mixtures were refluxed for overnight, cooled to room temperature, and extracted with chloroform. The organic layers were washed with brine, dried with MgSO_4_ and then evaporated. The obtained crude products were purified by column chromatography to give the requisite products **6**.

*5-(4,5-bis(4-(9H-Carbazol-9-yl)phenyl)thiophen-2-yl)thiophene-2-carbaldehyde* (**6a**). Yield: 80%; yellow solid; m.p: 140–145 °C; FT-IR: ʋ_max_ 1592, 1495, 1489, 1264, 737 cm^−1^; ^1^H-NMR (acetone-*d_6_*) δ 10.03 (s, 1H), 7.29-8.30 (m, 27H); ^13^C-NMR (acetone-*d_6_*) δ 205.5, 205.3, 183.1, 141.1, 131.4, 129.8, 127.6, 127.3, 126.3, 120.3, 110.0.

*5-(4,5-bis(4-(Diphenylamino)phenyl)thiophen-2-yl)thiophene-2-carbaldehyde* (**6b**). Yield: 85%; orange solid; m.p: 75–80 °C; FT-IR: ʋ_max_ 1424, 1265, 896, 730 cm^−1^; ^1^H-NMR (CDCl_3_) δ 9.86 (s, 1H), 7.66 (s, 1H), 7.38 (d, *J* = 11.0 Hz, 1H), 7.28 (d, *J* = 11.0 Hz, 1H), 6.90–7.27 (m, 28H); ^13^C-NMR (CDCl_3_) δ 182.7, 148.1, 148.0, 147.5, 147.4, 146.7, 142.2, 139.9, 139.6, 139.2, 133.4, 130.4, 129.5, 129.4, 129.1, 128.6, 127.4, 126.6, 124.9, 124.8, 124.7, 123.6, 123.5, 123.3, 123.2, 122.5. 

*5-(4,5-bis(4-(di-p-Tolylamino)phenyl)thiophen-2-yl)thiophene-2-carbaldehyde* (**6c**). Yield: 82%; orange solid; m.p: 76–80 °C; FT-IR: ʋ_max_ 1507, 1265, 896, 730 cm^−1^; ^1^H-NMR (CDCl_3_) δ 9.84 (s, 1H), 7.65 (s, 1H), 6.90–7.35 (m, 26H), 2.31 (s, 6H), 2.28 (s, 6H); ^13^C-NMR (CDCl_3_) δ 182.7, 148.6, 148.4, 146.9, 145.1, 144.9, 142.3, 139.7, 138.3, 133.1, 133.0, 132.9, 130.2, 130.1, 130.0, 128.9, 126.5, 125.1, 124.9, 122.4, 122.2, 122.0, 20.9.

#### 3.2.3. Typical Procedure for Synthesis of Compounds **9**

To stirred solutions of compounds **6** (1.47 mmol) and compound **8** (0.7 g, 2.20 mmol) in THF (30 mL) under an Ar atmosphere were added *t-*BuOK (0.3 g, 2.20 mmol) at 0 °C. The reaction mixtures were warmed to room temperature, stirred for 30 min and then extracted with chloroform. The organic layers were washed with brine, dried with MgSO_4_ and then evaporated. The obtained crude products were purified by column chromatography to give the requisite products **9**.

*9-(4-(3-(4-(9H-Carbazol-9-yl)phenyl)-5-(5-(2,2-diphenylvinyl)thiophen-2-yl)thiophen-2-yl)phenyl)-9H-carbazole* (**9a**). Yield: 55%; yellow solid; m.p: 135–140 °C; FT-IR: ʋ_max_ 1506, 1264, 730 cm^−1^; ^1^H-NMR (CDCl_3_) δ 6.90–8.20 (m, 38H); ^13^C-NMR (CDCl_3_) δ 140.1, 139.9, 131.0, 130.0, 129.8, 128.9, 128.6, 127.5, 127.2, 127.0, 126.1, 126.0, 124.8, 124.2, 123.5, 120.5, 120.4, 120.1, 109.9.

*4-(2-(4-(Diphenylamino)phenyl)-5-(5-(2,2-diphenylvinyl)thiophen-2-yl)thiophen-3-yl)-N,N-diphenyl-benzenamine* (**9b**). Yield: 53%; orange solid; m.p: 77–83 °C; FT-IR: ʋ_max_ 1599, 1502, 1453, 1264, 1231, 835, 700 cm^−1^; ^1^H-NMR (CDCl_3_) δ 6.90–7.52 (m, 42H); ^13^C-NMR (CDCl_3_) δ 147.7, 147.5, 144.0, 138.5, 134.8, 130.7, 130.3, 129.4, 129.3, 128.1, 127.5, 126.4, 124.6, 124.5, 123.8, 123.7, 123.2, 123.0, 122.0.

*4-(2-(4-(di-p-Tolylamino)phenyl)-5-(5-(2,2-diphenylvinyl)thiophen-2-yl)thiophen-3-yl)-N,N-di-p-tolyl-benzenamine* (**9c**). Yield: 58%; orange solid; m.p: 78–83 °C; FT-IR: ʋ_max_ 1592, 1495, 1489, 1264, 730 cm^−1^; ^1^H-NMR (CDCl_3_) δ 6.90–7.40 (m, 37H); ^13^C-NMR (CDCl_3_) δ 147.8, 147.5, 145.3, 144.9, 144.2, 138.8, 132.9, 132.6, 131.5, 130.8, 130.1, 130.0, 129.8, 128.5, 128.1, 127.2, 126.3, 124.9, 124.7, 123.5, 122.5, 121.8, 20.9. 

## 4. Conclusions

In summary, a series of green dopants based on 2,2-diphenylvinyl end-capped bithiophene and three different arylamine moieties, namely 9-phenylcarbazole, triphenylamine, and *N,N’*-di-(*p*- tolyl)benzeneamine, were successfully synthesized by Suzuki and Wittig coupling reactions. The strongest PL emitting compound with the 9-phenylcarbazole moiety has been used for fabricating an OLED device with yellowish green emission (CIE = 0.42, 0.54), maximum brightness and luminous efficiency of 5,100 cd/m^2^ and 2.56 cd/A, respectively.

## References

[1] Shirota Y. (2000). Organic materials for electronic and optoelectronic devices. J. Mater. Chem..

[2] Hung L.S., Chen C.H. (2002). Recent progress of molecular organic electroluminescent materials and devices. Mat. Sci. Eng. R.

[3] Lee M.-T., Yen C.-K., Yang W.-P., Chen H.-H., Liao C.-H., Tsai C.-H., Chen C.H. (2004). Efficient green coumarin dopants for organic light-emitting devices. Org. Lett..

[4] Ku S.-Y., Chi L.-C., Hung W.-Y., Yang S.-W., Tsai T.-C., Wong K.-T., Chen Y.-H., Wu C.-I. (2009). High-luminescence non-doped green OLEDs based on a 9,9-diarylfluorene-terminated 1,2,3-benzothiadiazole derivative. J. Mater. Chem..

[5] Wong K.-T., Wang C.-F., Chou C.H., Su Y.O., Lee G.-H., Peng S.-M. (2002). Synthesis and properties of novel thiophene-based conjugated homologues: 9,9-diphenylfluorene-capped oligothiophenes. Org. Lett..

[6] Gigli G., Inganӓs O., Anni M., Vittorio M.D., Cingolani R., Barbarella G., Favaretto L. (2001). Multicolor oligithiophene-based light-emitting diodes. Appl. Phys. Lett..

[7] Mazzeo M., Pisignano D., Favaretto L., Barbarella G., Cingolani R., Gigli G. (2003). Bright oligothiophene-based light emitting diodes. Synth. Met..

[8] Noda T., Ogawa H., Noma N., Shirota Y. (1999). Organic light-emitting diodes using a novel family of amorphous molecular materials containing an oligothiophene moiety as colour-tunable emitting materials. J. Mater. Chem..

[9] Noda T., Ogawa H., Noma N., Shirota Y. (1997). A novel family of amorphous molecular materials containing an oligothiophene moiety as colour-tunable emitting materials for organic electroluminescent devices. Adv. Mater..

[10] Noda T., Shirota Y. (1998). 5,5′-Bis(dimesitylboryl)-2,2′-bithiophene and 5,5′′-bis(dimesitylboryl)-2,2′:5′,2′′-terthiophene as a novel family of electron-transporting amorphous molecular materials. J. Am. Chem. Soc..

[11] Hock J., Cargill Thompson A.M.W., McCleverty J.A., Ward M.D. (1996). Some new dipyridyl and diphenol bridging ligands containing oligothienyl spacers, and their dinuclear molybdenum complexes: electrochemical, spectroscopic and luminescence properties. J. Chem. Soc., Dalton Trans..

[12] Field J.S., Haines R.J., Lakoba E.I., Sosabowski M.H. (2001). Novel diphenylphosphine derivatives of 2,2′-bithiophene, 2,2′:5′,2″-terthiophene, 2-(2′-thienyl)pyridine and 2,6-di-2′-thienylpyridine. Crystal structures of 5,5′-bis(diphenylphosphino)-2,2′-bithiophene, diphenyl{5-[6′-(diphenylphosphino)-2′-pyridyl]-2-thienyl}phosphine and 2,6-bis[5′-(diphenylphosphino)-2′-thienyl]pyridine. J. Chem. Soc., Perkin Trans. 1.

[13] Shirota Y., Kinoshita M., Noda T., Okumoto K., Ohara T. (2000). A novel class of emitting a morphous molecular materials as bipolar radical formants: 2-{4-[bis(4-methylphenyl)amino]phenyl}-5-(dimesitylboryl)thiophene and 2-{4-[bis(9,9-dimethylfluorenyl)amino]phenyl}-5-(dimesitylboryl)thiophene. J. Am. Chem. Soc..

[14] Kim J.-H., Jeon Y.-M., Lee H.S., Kim J.-W., Lee C.-W., Jang J.-G., Chang H.-J., Lee J.Y., Gong M.-S. (2008). New asymmetric monostyrylamine dopants for blue light-emitting organic electroluminescence device. Synth. Met..

[15] Kim S.O., Lee K.H., Kang S., Lee J.Y., Seo J.H., Kim Y.K., Yoon S.S. (2010). Highly efficient blue-light-emitting diodes based on styrylamine derivatives end-capped with a diphenylvinyl group. Bull. Korean Chem. Soc..

[16] Seong N.-C., Jeon Y.-M., Lim T.-H., Kim J.-W., Lee C.-W., Lee E.-J., Jang J.-G., Jang H.-J., Lee J.-Y. (2007). Organic light-emitting devices using new distyrylarylene host materials. Synth. Met..

[17] Guarin S.A.P., Bourgeaux M., Dufresne S., Skene W.G. (2007). Photophysical, crystallographic, and electrochemical characterization of symmetric and unsymmetric self-assembled conjugated thiopheno azomethines. J. Org. Chem..

[18] Rurack K., Spieles M. (2011). Fluorescence quantum yields of a series of red and near-infrared dyes emitting at 600–1000 nm. Anal. Chem..

[19] Liu Y., Wan Y., Guo H., Zhu M., Li C., Peng J., Zhu W., Cao Y. (2011). Synthesis and optoelectronic characterization of a monochromic red-emitting europium (III) complex containing triphenylamine-functionalized phenanthroline. J. Phys. Chem. C.

[20] Plater M.J., Jackson T. (2003). Polyaromatic amines. Part 3: synthesis of poly(diarylamino)styrenes and related compounds. Tetrahedron.

